# Evolutionary and Structural Features of the C2, V3 and C3 Envelope Regions Underlying the Differences in HIV-1 and HIV-2 Biology and Infection

**DOI:** 10.1371/journal.pone.0014548

**Published:** 2011-01-20

**Authors:** Helena Barroso, Pedro Borrego, Inês Bártolo, José Maria Marcelino, Carlos Família, Alexandre Quintas, Nuno Taveira

**Affiliations:** 1 Unidade dos Retrovírus e Infecções Associadas (URIA), Centro de Patogénese Molecular, Faculdade de Farmácia de Lisboa, Lisboa, Portugal; 2 Centro de Investigação Interdisciplinar Egas Moniz (CiiEM), Instituto Superior de Ciências da Saúde Egas Moniz, Caparica, Portugal; 3 Unidade de Tecnologia de Proteínas e Anticorpos Monoclonais, Instituto de Higiene e Medicina Tropical, Lisboa, Portugal; 4 Instituto de Medicina Molecular, Faculdade de Medicina de Lisboa, Lisboa, Portugal; City of Hope, United States of America

## Abstract

**Background:**

Unlike in HIV-1 infection, the majority of HIV-2 patients produce broadly reactive neutralizing antibodies, control viral replication and survive as elite controllers. The identification of the molecular, structural and evolutionary footprints underlying these very distinct immunological and clinical outcomes may lead to the development of new strategies for the prevention and treatment of HIV infection.

**Methodology/Principal Findings:**

We performed a side-by-side molecular, evolutionary and structural comparison of the C2, V3 and C3 envelope regions from HIV-1 and HIV-2. These regions contain major antigenic targets and are important for receptor binding. In HIV-2, these regions also have immune modulatory properties. We found that these regions are significantly more variable in HIV-1 than in HIV-2. Within each virus, C3 is the most entropic region followed by either C2 (HIV-2) or V3 (HIV-1). The C3 region is well exposed in the HIV-2 envelope and is under strong diversifying selection suggesting that, like in HIV-1, it may harbour neutralizing epitopes. Notably, however, extreme diversification of C2 and C3 seems to be deleterious for HIV-2 and prevent its transmission. Computer modelling simulations showed that in HIV-2 the V3 loop is much less exposed than C2 and C3 and has a retractile conformation due to a physical interaction with both C2 and C3. The concealed and conserved nature of V3 in the HIV-2 is consistent with its lack of immunodominancy *in vivo* and with its role in preventing immune activation. In contrast, HIV-1 had an extended and accessible V3 loop that is consistent with its immunodominant and neutralizing nature.

**Conclusions/Significance:**

We identify significant structural and functional constrains to the diversification and evolution of C2, V3 and C3 in the HIV-2 envelope but not in HIV-1. These studies highlight fundamental differences in the biology and infection of HIV-1 and HIV-2 and in their mode of interaction with the human immune system and may inform new vaccine and therapeutic interventions against these viruses.

## Introduction

Human Immunodeficiency Virus type 1 (HIV-1) infection affects more than 40 million individuals throughout the world. It is caused mainly by isolates belonging to group M. Within this group there are nine different subtypes named A to H, six subsubtypes (F1, F2, A1–A4) and at least thirty six recombinant forms named CRF01 up to CRF36 [1]. In contrast to the HIV-1 pandemic, HIV-2 is only prevalent in West Africa where it seems to have been present since the 1940s [2]. In Europe infection with HIV-2 remains rare (2–3% of all AIDS cases), being observed mainly in France and Portugal [3], [4], [5]. Eight different HIV-2 groups named A through H have been reported but only groups A and B cause human epidemics [6], [7], [8], [9]. Isolates from group A are, however, responsible for the vast majority of HIV-2 infections worldwide [10].

For reasons that are still not clear, HIV-1 and HIV-2 infections lead to very different immunological and clinical outcomes. In contrast to HIV-1 infected patients, the majority of HIV-2-infected individuals have reduced general immune activation, normal CD4+ T cell counts, low or absent viremia and absence of clinical disease [11], [12], [13], [14]. This may be related with a more effective immune response produced against HIV-2. In fact, most HIV-2 infected individuals have strong cytotoxic responses to Env and Gag proteins and raise autologous and heterologous neutralizing antibodies [3], [15], [16], [17], [18]. The attenuated course of HIV-2 infection compared to HIV-1 has also been associated to a lower state of immune activation, which may be related to the immunosuppressive activity of the C2-V3-C3 envelope region [19], [20], [21]. Similar immunosuppressive activity has not been found in the homologous C2-V3-C3 region in the HIV-1 envelope [19]. Finally, the transmission rate of HIV-2 is also significantly lower than that of HIV-1 and this has been associated with the low or absent viremia found in most HIV-2 patients [22], [23].

The HIV-1 Env glycoprotein is a trimer on the virion surface with extensive N-linked glycosylation that effectively shields many conserved epitopes from antibody recognition [24]. It is composed of trimers of a surface (SU) glycoprotein with a molecular weight of 120–125 kDa (gp120–125) that is bound to a transmembrane (TM) glycoprotein with 36–41 kDa (gp36–41). SU can be divided into five hipervariable regions, named V1 to V5, bordered by five conserved regions, named C1 to C5. The C2 and C3 regions associate to form the CD4 binding site such that mutations in amino acid at positions 267Q in C2 and 368R in C3 abrogate gp120 binding to CD4 [25], [26]. In HIV-1, V3 is one of the most important determinants of viral tropism and co-receptor usage [27], [28]. This region also contains major antigenic and neutralizing epitopes in HIV-1 which are well exposed upon CD4-binding [29], [30], [31], [32], [33], [34], [35]. Although still debatable, the V3 region in HIV-2 may also contain broadly neutralizing epitopes [36], [37], [38], [39], [40], [41], [42]. However, in contrast to HIV-1, the V3 and flanking C2 and C3 regions are not immunodominant in HIV-2 infected patients [43], [44], [45], [46]. Moreover, it remains to be determined whether these regions are exposed or concealed in the envelope complex of primary isolates of HIV-2.

In HIV-1 infection escape from antibody neutralization occurs frequently and is the major driving force of the molecular evolution of the envelope glycoproteins [47], [48]. Not surprisingly, codons under diversifying selection (positive selection) seem to be clustered mostly in the hypervariable V1/V2 and V3 regions that contain important and accessible neutralizing targets [49], [50]. The impact of the neutralizing antibody response in the *in vivo* evolution of the HIV-2 Env is currently unknown.

The present study was designed to identify molecular and evolutionary features of the C2, V3 and C3 regions in HIV-1 and HIV-2 infected patients that could be related with their different immunological and clinical outcomes. We describe some potentially important differences in the genetic constitution, molecular evolution and conformation of the C2, V3 and C3 regions in HIV-1 and HIV-2 that provide new insights into their function and may inform the design of HIV vaccines.

## Results

### HIV-1 is significantly more variable in the envelope C2, V3 and C3 regions than HIV-2

We compared the inter-patient genetic diversity of HIV-1 and HIV-2 in two different datasets: HIV-1 group M (all subtypes) and HIV-2 group A sequences from all over the world (Control dataset composed of reference sequences) and newly derived HIV-1 and HIV-2 sequences obtained from Portuguese (PT) patients. Phylogenetic analysis showed that HIV-1 sequences circulating in Portugal belong to different subtypes and recombinant forms (Figure S1A). Forty five sequences were subtype B and six belonged to the recombinant form CRF14_BG. Subtypes G (4 sequences) and C (2), sub-subtype F1 (2), and CRF02_AG (1) were also found. Regarding HIV-2, all sequences from Portugal clustered together within group A (Figure S1B). Collectively, these results are consistent with previous studies showing a highly complex HIV epidemics in Portugal caused exclusively by HIV-2 group A and different subtypes of HIV-1 group M [51], [52], [53], [54]. Nucleotide diversity between HIV-1 viruses found in Portugal was significantly higher compared to HIV-2 (mean number of substitutions per site, 0.336, 95%CI [0.329; 0.342] *vs* 0.239, [0.236; 0.243], P<0.0001). Similar results were found for the HIV-1 and HIV-2 Control datasets (Table S1). Hence, we conclude that HIV-1 is genetically more diverse than HIV-2 in the envelope region comprising C2, V3 and C3.

**Figure 1:**
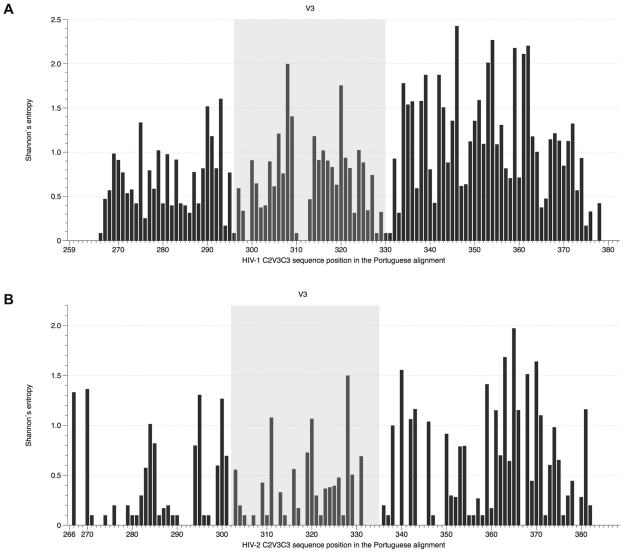
Shannon's entropy of individual amino acids in the C2, V3 and C3 envelope regions in HIV-1 and HIV-2. (A) HIV-1 alignment (PT dataset), sites were numbered according to codon *env* position of HIV-1 HXB2 reference strain; (B) HIV-2 alignment (PT dataset), sites were numbered according to codon *env* position of HIV-2 ALI reference strain.

Amino acid diversity in the C2, V3 and C3 regions of HIV-1 and HIV-2 were compared by calculating Shannon's entropy [55]. Mean entropy values for the three regions were significantly higher in HIV-1 than in HIV-2 both in PT (0.794 *vs* 0.409, P<0.0001) and Control datasets (0.702 *vs* 0.353, P<0.0001) confirming that these regions are more variable in HIV-1 than in HIV-2. Entropy was also significantly higher in HIV-1 than in HIV-2 in each separate region (C2, P<0.05; V3, P<0.005; C3, P<0.0005) of PT sequences. The region with higher mean entropy was C3 in both viruses (1.031, 95%CI [0.845, 1.217] for HIV-1 *vs* 0.534, 95%CI [0.378, 0.689] for HIV-2, P<0.0005) followed by V3 (0.674, [0.506, 0.841]) and C2 (0.574, [0.427, 0.721]) in HIV-1 and C2 (0.326, [0.175, 0.477]) and V3 (0.304, [0.176, 0.433]) in HIV-2 (Figure 1). Comparable results were obtained for the Control datasets but in this case V3 was the least entropic region both in HIV-1 and HIV-2 (Table S1 and Figure S2). Not surprisingly, amino acids with higher entropy (values above 1) were primarily located in the C3 region of both viruses and there were more highly entropic amino acids in C3 in HIV-1 than in HIV-2 both in the PT and Control datasets (PT dataset: 51.9% in HIV-1 *vs* 24.5% in HIV-2; Control dataset: 35.3% in HIV-1 *vs* 20.8% in HIV-2). Notably, the amino acids in V3 that are related with co-receptor usage, positions 11/25 in HIV-1 (codons 306/320) [56], [57] and possibly positions 18/19/27 in HIV-2 (codons 319/320/328) [17], [58], had a high entropy score in both viruses.

**Figure 2:**
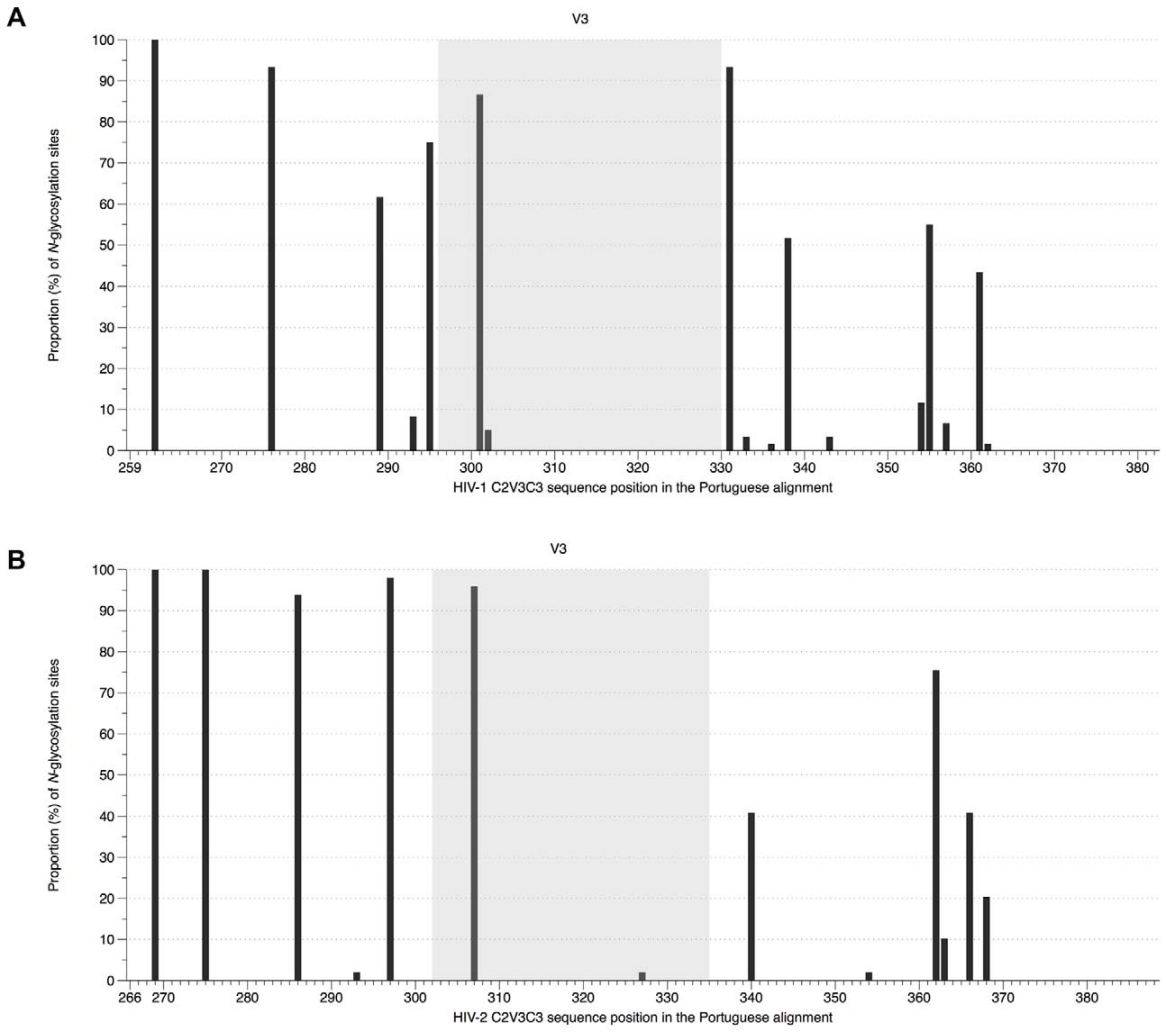
Frequency of *N*-glycosylation sites in the C2, V3 and C3 envelope regions in HIV-1 and HIV-2. (A) HIV-1 alignment (PT dataset), sites were numbered according to codon *env* position of HIV-1 HXB2 reference strain; (B) HIV-2 alignment (PT dataset), sites were numbered according to codon *env* position of HIV-2 ALI reference strain.

The mean number of potential *N*-linked glycosylation sites both in HIV-1 and HIV-2 sequences from Portugal was 7 (range: 4–9 in HIV-1; 5–9 in HIV-2). The most conserved glycosylation sites were located in C2 in both viruses (Figure 2). Nonetheless, in this region, there were four highly conserved glycosylation sites in HIV-2 (present in ≥80% of strains) and only two such sites in HIV-1. With the exception of the highly conserved site located in the beginning of C3 in HIV-1, glycosylation sites found in C3 varied from strain to strain in number and location, this being more evident in HIV-1 than in HIV-2. In V3 there were two highly conserved glycosylation sites in both viruses. Similar observations were made for HIV-1 and HIV-2 sequences in the Control datasets (Table S1 and Figure S3).

### Selective pressures act differently in HIV-1 and HIV-2

We have recently found that HIV-2 displays a faster evolutionary rate in the envelope gp125 and C2-V3-C3 region than HIV-1 in patients with chronic and advanced disease [52], [59]. The faster evolutionary rate in HIV-2 was more pronounced in synonymous sites than in non-synonymous sites suggesting a weaker positive selection in HIV-2 than in HIV-1. To investigate this possibility, we analysed diversifying selection in the C2-V3-C3 region of both viruses using codon-based models of molecular evolution. Firstly, we estimated the ratio of non-synonymous and synonymous substitution rates (dN/dS ratio) averaged over all sites. For HIV-1 sequences from Portugal dN/dS ratio was 0.703, 95%CI [0.668, 0.740]; for HIV-2 it was 0.451, [0.419, 0.484]. Similar values were obtained for the Control alignments (Table S1). These results are consistent with the higher degree of genetic conservation of the C2, V3 and C3 regions in HIV-2.

**Figure 3:**
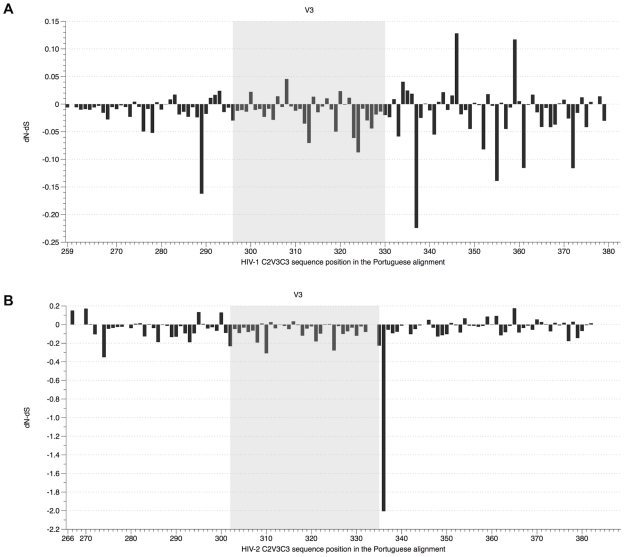
Positive selection in the C2, V3 and C3 envelope regions in HIV-1 and HIV-2. dN-dS values were estimated by FEL and scaled by the total codon tree length. (A) HIV-1 alignment (PT dataset), sites were numbered according to codon *env* position of HIV-1 HXB2 reference strain; (B) HIV-2 alignment (PT dataset), sites were numbered according to codon *env* position of HIV-2 ALI reference strain.

Site-by-site analysis revealed that diversifying selection is unevenly distributed along the studied region between the two viruses (PT, P<0.001; Controls, P<0.001) (Figures 3 and S4). For HIV-2 sequences from the PT dataset, there were between 7 and 9 positively selected (PS) sites depending on the method that was used (SLAC/FEL/REL) while for HIV-1 the number of sites ranged from 7 to 17 (Table 1). Taking into account only sites that were selected by at least two methods, HIV-2 had a total of 7 PS sites whereas in HIV-1 there were 9 sites. The sites were distributed as follows: in C2 there were 3 sites in HIV-2 and 2 in HIV-1; in V3 there were 2 sites in HIV-1, and no sites in HIV-2; in C3 there were 4 sites in HIV-2 and 5 in HIV-1, including one codon within the CD4 binding site (codon 378 in HIV-1) and two in the α2-helix (codons 343 and 346) [60]. In Control data sets the number of PS sites was slightly lower but they were similarly distributed, with the exception of the V3: 1 PS site in HIV-2, but no sites in HIV-1 (Tables S1 and S2). Importantly, we found that when compared to HIV-1, positive selection was stronger in HIV-2 in most sites (Tables 1 and S2).

**Table 1:** Positively selected sites detected by SLAC, FEL, REL and/or IFEL in HIV-1 and HIV-2 *env* C2, V3 and C3 regions^1^.

HIV-1										HIV-2									
Region	Codon	SLAC		FEL		REL		IFEL		Region	Codon	SLAC		FEL		REL		IFEL	
**C2**	**283**	**0.249**	(0.083)	**0.017**	(0.027)	**0.282**	(0.950)	−0.007	(0.237)	**C2**	**267**	**1.805**	(<0.001)	**0.151**	(<0.001)	**1.007**	(1.000)	**0.147**	(0.004)
	291	**0.252**	(0.096)	0.011	(0.334)	0.005	(0.277)	0.008	(0.648)		**270**	**1.561**	(0.003)	**0.171**	(<0.001)	**0.892**	(1.000)	**0.179**	(0.010)
	292	**0.269**	(0.063)	0.017	(0.167)	0.547	(<0.001)	0.014	(0.419)		295	**1.049**	(0.051)	**0.134**	(0.095)	0.070	(0.910)	0.109	(0.316)
	293	**0.401**	(0.066)	0.024	(0.230)	**0.924**	(0.972)	**0.063**	(0.050)		300	0.787	(0.210)	**0.130**	(0.077)	0.714	(<0.001)	0.131	(0.109)
**V3**	300	**0.335**	(0.079)	**0.022**	(0.022)	0.219	(0.846)	**0.016**	(0.093)	**V3**	331	−0.312	(0.859)	0.020	(0.591)	**0.193**	(0.987)	0.069	(0.390)
	306	0.312	(0.106)	0.014	(0.541)	**0.947**	(0.984)	−0.004	(0.867)										
	**308**	**0.619**	(0.008)	**0.046**	(0.065)	**0.869**	(0.973)	**0.094**	(0.012)										
	314	**0.314**	(0.052)	0.014	(0.291)	0.163	(0.178)	−0.001	(0.971)										
	317	**0.301**	(0.057)	0.011	(0.401)	0.192	(0.140)	0.005	(0.749)										
**C3**	332	0.124	(0.267)	0.009	(0.341)	−0.432	(<0.001)	**0.031**	(0.093)	**C3**	346	0.478	(0.236)	0.051	(0.173)	**1.008**	(1.000)	0.050	(0.397)
	**334**	**0.543**	(0.004)	**0.041**	(0.027)	**1.142**	(0.997)	**0.065**	(0.024)		351	0.207	(0.298)	**0.018**	(0.087)	0.114	(<0.001)	0.000	(1.000)
	335	**0.458**	(0.010)	0.025	(0.109)	0.893	(0.936)	0.035	(0.147)		**354**	**0.689**	(0.047)	**0.067**	(0.016)	**0.988**	(1.000)	0.000	(1.000)
	336	**0.452**	(0.058)	0.019	(0.583)	0.817	(0.907)	0.011	(0.743)		**361**	**0.887**	(0.035)	**0.093**	(0.011)	**0.988**	(1.000)	0.016	(0.693)
	343	**0.405**	(0.060)	0.022	(0.109)	**0.885**	(0.989)	0.017	(0.370)		364	−0.085	(0.704)	0.014	(0.690)	**0.130**	(0.983)	−0.069	(0.072)
	345	**0.392**	(0.024)	0.016	(0.330)	0.492	(0.657)	**0.070**	(0.018)		365	**1.074**	(0.089)	**0.175**	(0.070)	0.463	(0.561)	−0.056	(0.626)
	**346**	**1.080**	(<0.001)	**0.128**	(<0.001)	**0.945**	(0.982)	**0.281**	(<0.001)		378	**0.415**	(0.088)	**0.030**	(0.043)	0.116	(<0.001)	0.029	(0.119)
	348	**0.270**	(0.096)	0.011	(0.664)	1.183	(<0.001)	−0.005	(0.899)										
	353	0.319	(0.143)	0.018	(0.476)	−0.118	(0.347)	**0.120**	(0.035)										
	**359**	**0.558**	(0.022)	**0.117**	(<0.001)	**0.882**	(1.000)	**0.214**	(0.001)										
	363	0.169	(0.295)	0.017	(0.279)	**0.860**	(0.995)	0.003	(0.900)										
	378	**0.244**	(0.021)	**0.014**	(0.020)	0.217	(<0.001)	0.000	(1.000)										

1 PT dataset.

Codon – codons selected under 10% level of significance (SLAC, FEL and IFEL) or above a Bayes Factor of 50 (REL) and numbered according to codon *env* position of HIV-1 HXB2 for HIV-1 dataset or of HIV-2 ALI for HIV-2 dataset. Codons selected simultaneously by SLAC, FEL and REL methods are bold and underlined.

SLAC, FEL and IFEL – the first numbers are the dN-dS difference for each site scaled by the total codon tree length, the numbers in parenthesis show P-values for corresponding test of non-synonymous rate being superior to synonymous rate; REL - the first numbers are the expected posterior dN-dS difference for each site scaled to the total codon tree length, the number in parenthesis show the posterior probability of non-synonymous rate being superior to synonymous rate; Bold dN-dS differences correspond to significant P-values or posterior probabilities.

The comparison of diversifying selection between terminal and internal branches of the phylogenetic trees revealed two distinct profiles for HIV-1 and HIV-2. Firstly, non-synonymous substitution rates were significantly different between the internal nodes and the tips of the tree in all datasets: PT, P = 0.002 for HIV-2 and P = 0.011 for HIV-1; Controls, P<0.001 and P = 0.004 (data not shown). Stronger selection was in general found at codons selected simultaneously at the tips and the external branches of the HIV-1 and HIV-2 trees. Importantly, however, only 2 of the 7 sites (29%) detected in terminal branches of PT HIV-2 tree were also under positive selection along the internal branches (codons 267 and 270 in C2). In contrast, for HIV-1 most positively selected sites (6/9, 67%) were present both in the internal and the terminal branches. In Control datasets these percentages were 43% for HIV-2 and 71% for HIV-1 (Table S2). These results suggest that natural selection affects less the transmission fitness of HIV-1 than HIV-2.

### Structure and solvent accessibility of V3 differ in HIV-1 and HIV-2

**Figure 4:**
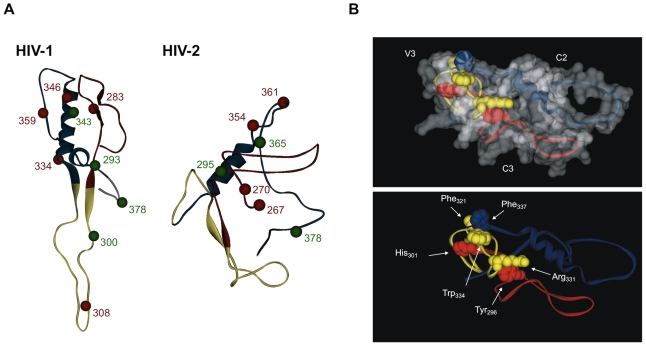
Conformational structure of C2, V3 and C3 envelope regions in HIV-1 and HIV-2. The conformational structure of consensus amino acid sequences derived from the PT datasets was obtained by homology modeling as indicated in Material and Methods. In the schematics, C2 is shown in red, V3 in yellow and C3 in blue. (A) Balls represent the amino acids under positive selection. The red balls represent codons selected simultaneously by SLAC, FEL and REL methods, while green balls stand for codons selected by at least two of these methods; (B) Model structure showing the predicted interactions between V3, C2 and C3 in HIV-2 gp125. The non-covalent interaction involves residues Tyr296 and His301 in C2 binding, respectively, to Arg331 and Trp334 in V3, and Phe337 in C3 binding to Phe321 in V3.

A model of the structure of the C2-V3-C3 region was built for HIV-1 and HIV-2 based on the atomic coordinates of the HIV-1 gp120 and SIV gp120 using consensus sequences from both the PT and Control HIV-1 and HIV-2 alignments. For HIV-1, the structures of PT and Control sequences were almost identical having only a slight difference in V3, which presents less regular secondary structure in the PT sequence (Figure S5). For HIV-2, the structures of PT and Control sequences were identical. The structure of the C2-V3-C3 region was however markedly different between HIV-1 and HIV-2, the most striking differences being the significant retraction of the V3 loop in HIV-2 and its potential interaction both with C2 and C3 (Figure 4A). Identical results were obtained when comparing the HIV-1 and HIV-2 control sequences (Figure S6). The predicted non-covalent interaction between V3, C3 and C3 in HIV-2 involves residues Tyr296 and His301 in C2 binding, respectively, to Arg331 and Trp334 in V3, and Phe337 in C3 binding to Phe321 in V3 (Figure 4B).

**Figure 5:**
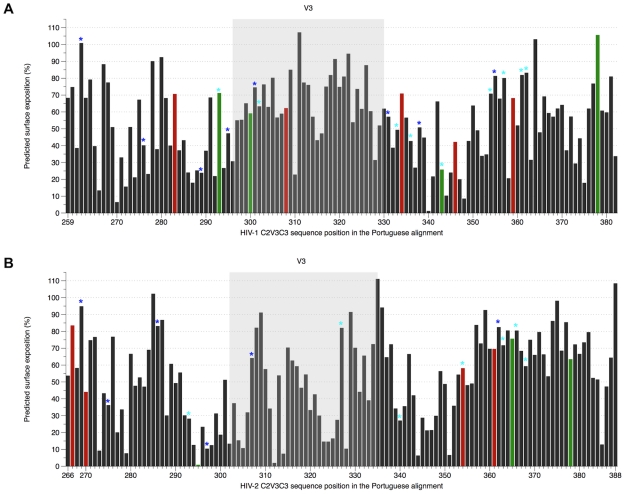
Solvent accessible surface area, positive selection and potential *N*-glycosylation sites in C2-V3-C3 region. (A) HIV-1 alignment (PT dataset), sites were numbered according to codon *env* position of HIV-1 HXB2 reference strain; (B) HIV-2 alignment (PT dataset), sites were numbered according to codon *env* position of HIV-2 ALI reference strain. Coloured bars represent the amino acids under positive selection and have the same colours (red and green) as the corresponding positions (balls) highlighted in Figure 4A. The dark blue stars over the bars correspond to potential *N*-glycosylation sites conserved along the alignment (present in ≥50% of strains), whereas the light blue stars represent sites only present in less than 50% of sequences.

The solvent accessibilities of amino acid residues were also calculated for both models (Figure 5). As expected, both in HIV-1 and HIV-2 most PS sites and *N*-glycans had at least 50% surface exposure. In HIV-2, 8 out of 37 (22%) amino acids in C2, 8/34 (24%) in V3 and 19/53 (36%) in C3 were highly exposed (≥70% solvent accessibility) whereas in HIV-1 these were 9/37 (24%), 15/35 (43%) and 10/52 (19%), respectively. Consistent with the high exposure of the V3 region in HIV-1, the two amino acids at positions 306 and 320 involved in binding to co-receptors were well exposed (≥50% solvent accessibility). In contrast, in HIV-2, among amino acids 319/320 and 328 in V3 loop potentially involved in co-receptor binding, only 319 was relatively well exposed. Despite the potential interaction between V3 and C3 (Figure 4B), the overall exposition of C3 was higher in HIV-2 than in HIV-1. Thus, for instance, 42% (5/12) of the residues in C3 that may contribute for the formation of the CD4-binding site (positions 377–388) in HIV-2 showed high solvent accessibility. In HIV-1 only 3 out of 16 (19%) amino acids with similar function (positions 367–382) were highly exposed. Similar results were obtained when comparing the HIV-1 and HIV-2 control sequences (Figure S7).

## Discussion

To investigate the molecular and structural features underlying the differences in HIV-1 and HIV-2 biology and human infection, we have analysed the C2-V3-C3 envelope region from a significant number of HIV-1 and HIV-2 infected patients living in Portugal and worldwide. HIV-2 sequences from Portugal belonged to group A and the majority of HIV-1 sequences belonged to subtype B (75%) followed by subtypes G, C and F, CRF02_AG and CRF14_BG. Collectively, these results are consistent with previous studies showing a highly complex HIV epidemic in Portugal caused by HIV-2 group A and different subtypes of HIV-1 group M [44], [51], [52], [53], [54], [61], [62].

Genetic distances and amino acid diversity between HIV-1 viruses were significantly higher compared to HIV-2. This was surprising since at the individual level HIV-2 displays a similar [52] or even faster evolutionary rate than HIV-1 in the C2-V3-C3 region [59]. The more pronounced evolutionary rate in synonymous sites than in non-synonymous sites in HIV-2 [59] together with the rare escape of this virus from autologous neutralizing antibodies [17], suggested that the lower amino acid diversity in HIV-2 could be related with a weaker positive selection or even with negative selection [62]. This was not the case however since most sites in C2 and C3 were under stronger positive selection in HIV-2 than in HIV-1. The C3 region of HIV-1 is antibody accessible [63] and is subject to diversifying selection because it is a major neutralizing target [64], [65], [66], [67]. Therefore, the high level of positive selection detected in C3 together with its high solvent exposure strongly suggests that this region is also antibody accessible in HIV-2 and might be a major neutralizing domain.

Strength of selection was significantly different between internal and external branches of the HIV-1 and HIV-2 phylogenetic trees. This is expected in populations of highly variable RNA viruses and implies that non-synonymous substitutions can be highly deleterious [68], [69]. In HIV-1, most of the codons selected in the tips of the tree were also under selection along the internal branches, indicating that adaptation in these sites is occurring at the host and population levels [68]. In contrast, most adaptive mutations in HIV-2 were only found in the tips of the tree indicating that they are recent maladaptive substitutions that are transitory at the population level [68], [70]. Thus, in contrast to HIV-1, diversification of C2 and C3 in HIV-2 seems to have a dominant negative effect on viral fitness and transmission. This data suggests that one possible consequence of the unexpectedly high evolutionary rate of HIV-2 at the patient level can be the frequent accumulation of deleterious mutations and production of defective viruses [52], [59], [71]. A high frequency of defective viruses in HIV-2 infected individuals could explain the poor replication of this virus *in vivo* as well as its very low transmissibility.

Unlike in HIV-1, the V3 loop in HIV-2 always presented the lower amino acid diversity. This result might be a consequence of significant structural and conformational constraints due to its role in preventing chronic and disruptive immune activation [20] and in co-receptor binding [58]. On the other hand, these results imply that the V3 loop is not well exposed in the HIV-2 envelope complex *in vivo*. Indeed, by computer modelling simulations we show that in HIV-2 the V3 loop is much less exposed than C2 and C3 and likely has a retractile conformation due to non-covalent interaction both with C2 and C3. In contrast, HIV-1 had, as previously found, an extended and highly accessible V3 loop [66], [67], [72]. Such conformation is entirely consistent with its immunodominant and neutralizing nature and with its crucial role in HIV-1 co-receptor binding and tropism [33], [34], [35], [73], [74], [75]. Conversely, the concealed nature of V3 in the HIV-2 envelope complex implies that this region may not be immunodominant in HIV-2 infection. Indeed, a significant number of HIV-2 patients do not raise antibodies against the V3 loop [43] or against a polypeptide comprising the C2, V3 and C3 regions [45]. Thus, the occlusion of V3 in the HIV-2 envelope complex may prevent it from over immune recognition and associated sequence changes thereby preserving its crucial functions in viral entry. It has been shown that removal or antigenic dampening of the HIV-1 V3 loop redirects the neutralizing immune response to other epitopes of the Env protein that otherwise would be non-neutralizing or non-antibody responsive [33], [76], [77], [78]. In this context, the occluded nature of the V3 region in the HIV-2 envelope complex might favour a more effective production of broadly neutralizing antibodies targeting other regions in gp125 such as the C2, V1, V2, V4 and C5 regions [37], [38], [39], [79].

In conclusion, the C2 and C3 regions are well exposed in the HIV-2 envelope complex and are under strong diversifying selection suggesting that, like in HIV-1, they may harbour neutralizing epitopes. However, extreme diversification of C2 and C3 in HIV-2 seems to be deleterious for the virus and prevent its transmission. On the other hand, V3 is highly conserved in HIV-2 and is concealed within the envelope complex, possibly due to a physical interaction with C2 and C3. In contrast, V3 is highly exposed and variable in HIV-1 which is consistent with its immunodominant and neutralizing properties. Collectively, we identify significant structural and functional constrains to the diversification and evolution of C2, V3 and C3 in the HIV-2 envelope but not in HIV-1. These studies highlight fundamental differences in the biology and infection of HIV-1 and HIV-2 and in their mode of interaction with the human immune system and may inform new vaccine and therapeutic interventions against these viruses.

## Materials and Methods

### Amplification, cloning and sequencing of HIV-1 and HIV-2 viruses from Portugal

Portuguese (PT) samples were collected from HIV infected patients, followed in hospitals in the North and South of Portugal and presenting different clinical stages of infection and CD4+ T-cell counts. HIV-2 samples were collected between 1997 and 2005 from 49 patients, some of whom were infected in late-1970s [52], [80]. HIV-1 samples were collected from 60 patients between 1993 and 1998.

Proviral DNA was extracted from uncultured PBMCs, or viral genomic RNA was extracted from plasma and reverse transcribed. A nested PCR technique was used to amplify a 373 bp HIV-2 C2-V3-C3 *env* gene region and a 409 pb HIV-1 C2-V3-C3 *env* region as described elsewhere [62], [81]. PCR products were sequenced using the BigDye Terminator Cycle sequencing kit (Applied Biosystems) and an automated capillary sequencer (ABI PRISM 310, Applied Biosystems). Newly derived HIV-1 sequences from Portugal have been assigned GenBank accession numbers: EU335962 - EU335903. Newly derived HIV-2 sequences from Portugal have been assigned GenBank accession numbers: AY913773-AY913794, AY649545-AY649554 and GU591163.

Additionally, 16 HIV-2 consensus sequences from a previous publication [52] were also included in this study. The samples used to obtain these consensus sequences were: 03PTHCC1, 03PTHCC2, 03PTHCC4, 03PTHCC5, 03PTHCC7, 03PTHCC8, 03PTHCC12, 05PTHCC13, 03PTHCC14, 03PTHCC17, 03PTHCC19, 03PTHSM2, 05PTHSM3, 03PTHSM7, 03PTHSM9 and 03PTHSM10.

### Control datasets

As Control datasets to this study, HIV-1 group M (all subtypes) reference sequence alignment (94 sequences) was obtained from the Los Alamos HIV database (http://www.hiv.lanl.gov/). HIV-2 group A reference sequence alignment was also obtained from the Los Alamos HIV database. Additional C2-V3-C3 sequences derived from group A primary isolates were retrieved from the Los Alamos Database adding to a total of 59 HIV-2 Control sequences. Both control alignments are available as supplementary information (Alignment S1 and S2).

### Molecular and phylogenetic analysis

Nucleotide sequences were aligned using ClustalX 1.8 [82]. Maximum likelihood analyses were performed using the best-fit models of molecular evolution estimated by Modeltest [83]. These were GTR+G+I [84] for the PT HIV-2 dataset and TVM+G+I for PT HIV-1 and for HIV-1 and HIV-2 Control datasets [85].

Evolutionary distances were estimated under these models using PAUP version 4.0 [86]. Tree searches were also conducted in PAUP version 4.0 using either nearest-neighbor interchange (NNI) or subtree pruning-regrafting (SPR) heuristic strategies, with bootstrap resampling. All positions containing gaps and missing data were eliminated from the dataset. In the final datasets there were a total of 369 nucleotide positions in PT HIV-2 and 372 positions in PT HIV-1 alignments, and 369 positions in HIV-2 and HIV-1 Control alignments. Both alignments were tested for recombination with the Single Breakpoint Recombination (SBP) tool [87] in the DATAMONKEY web-server [88]; evidence for recombination, inferred by the small sample AIC score, was only found for HIV-1 Control dataset. Thus, when appropriate, a multiple partition dataset was used for HIV-1 Control analysis. Detection of N-linked glycosylation sites was performed with Glycosite [89]. The entropy at each position in protein alignment was measured with Shannon's entropy [55].

### Tests for codon selection

Selection pressures over the HIV-1 and HIV-2 C2-V3-C3 regions were examined with the HYPHY software package [90] and the DATAMONKEY web-server [88]. All estimations were performed using the MG94 codon substitution model [91] crossed with the nucleotide substitution model previously selected with Modeltest, GTR for PT HIV-2 and TVM for PT HIV-1 and Control alignments. To understand if selection pressure within a host is different from selection for transmission among hosts, non-synonymous substitutions were compared between terminal and internal branches of the phylogenetic tree, with the TestBranchDNDS.bf batch file in HyPHy, as described elsewhere [92].

Four different approaches were used to identify codons under selection: single-likelihood ancestor counting (SLAC), fixed-effects likelihood (FEL), internal fixed effects likelihood (IFEL) and relaxed-effects likelihood (REL) methods [68], [93]. While SLAC, FEL and REL detect sites under selection at the external branches of the phylogenetic three, IFEL identifies such sites only along the internal branches. To classify a site as positively or negatively selected the cut-off P-value was 10% for SLAC, FEL and IFEL. For REL, codons under selection were detected with a cut-off value for the Bayes factor of 50. Since SLAC, FEL and IFEL can estimate site-specific ratios of non-synonymous and synonymous substitutions rates (dN/dS ratios) as undefined or infinite due to dS  = 0, we reported dN-dS values instead, which were scaled by the total codon tree length to allow a better comparison between the two datasets. A multiple partition dataset was used for the identification of codons under selection in HIV-1 Control analysis. Site-by-site variation of synonymous substitution rates can bias estimations of codon's diversifying selection [94]. Although all four methods described above model for this variation, variation of synonymous rates from codon to codon in each dataset was tested with the dNdSRateAnalysis.bf batch file in HyPHy, as described elsewhere [92]. Finally, comparison of the dN/dS distribution rates and the strength of selection between the HIV-1 and HIV-2 alignments, was performed with dNdSDistributionComparison.bf batch file also in HyPHy, as described elsewhere [92].

### Molecular modelling and calculation of solvent accessible surfaces

Consensus amino acid sequences were derived for the different HIV-1 and HIV-2 datasets. Structural models of HIV-1 and HIV-2 C2-V3-C3 were produced with SWISS-MODEL homology modelling server in project mode resorting to Swiss-Pdb Viewer (DeepView) version 4.0, using PDB file 2B4C (from HIV-1 JR-FL gp120) for HIV-1, and PDB file 2BF1 (from SIV gp120) for HIV-2 as templates [95], [96], [97], [98]. Accelrys Discovery Studio Visualizer 2.5 [99] was used to produce three dimensional images of the models obtained. Solvent accessible surface area in Å^2^ was calculated by Gerstein's calc-surface software on UCSF Chimera [100], [101] with a probe size of 1.4 Å. All atoms in the input PDB file were included in the calculation. The solvent accessible surface data was normalized dividing each amino acid residue solvent accessible surface value added by the solvent accessible surface value of the corresponding amino acid residue (X) in the tripeptide Gly-X-Gly. The inter-chain H-Bonds formed by HIV-2 V3 with C2 and C3 were calculated with H-Bond Finder software on UCSF Chimera [100], [101] with a probe size of 1.4 Å. All atoms in the input PDB file were included in the calculation.

### Statistical analysis

Statistical analyses were performed using GraphPad Prism version 4.0c for Macintosh (GraphPad Software, 2005, San Diego, California, USA, www.graphpad.com) with a level of significance of 5%. Non-parametric Mann-Whitney U test was used to compare Shannon's entropy values and nucleotide distances.

## Supporting Information

Figure S1 Genotyping HIV-1(A) and HIV-2 (B) by maximum-likelihood phylogenetic analysis. The phylogenetic trees were constructed using the SPR heuristic search strategy and 1000 bootstrap replications, with reference sequences from HIV-1, under the TVM+G+I evolutionary model (A) and with reference sequences from HIV-2, under the GTR+G+I evolutionary model (B). The bootstrap values (above 50%) supporting the internal branches are shown. The scale bar represents evolutionary distances in substitutions per site.
(0.21 MB PDF)

Figure S2 Shannon's entropy of individual amino acids in the C2, V3 and C3 envelope regions in HIV-1 and HIV-2. (A) HIV-1 alignment (Control dataset), sites were numbered according to codon env position of HIV-1 HXB2 reference strain; (B) HIV-2 alignment (Control dataset), sites were numbered according to codon env position of HIV-2 ALI reference strain.
(0.96 MB TIF)

Figure S3 Frequency of N-glycosylation sites in the C2, V3 and C3 envelope regions in HIV-1 and HIV-2. (A) HIV-1 alignment (Control dataset). Sites were numbered according to codon env position of HIV-1 HXB2 reference strain. (B) HIV-2 alignment (Control dataset). Sites were numbered according to codon env position of HIV-2 ALI reference strain.
(0.58 MB TIF)

Figure S4 Positive selection in the C2, V3 and C3 envelope regions in HIV-1 and HIV-2. dN-dS values were estimated by FEL and scaled by the total codon tree length. (A) HIV-1 alignment (Control dataset). Sites were numbered according to codon env position of HIV-1 HXB2 reference strain. (B) HIV-2 alignment (Control dataset). Sites were numbered according to codon env position of HIV-2 ALI reference strain.
(0.53 MB TIF)

Figure S5 Superimposition of the conformational structures generated by homology modelling of Portuguese and Control C2, V3 and C3 regions of HIV-1 and HIV-2. In the schematics, Portuguese structures are represented in red, and Control structures are in blue.
(0.78 MB TIF)

Figure S6 Conformational structure of C2, V3 and C3 envelope regions in HIV-1 and HIV-2. The conformational structure of consensus amino acid sequences derived from the Control datasets was obtained by homology modeling as indicated in “Materials and Methods.” In the schematics, C2 is shown in red, V3 in yellow, and C3 in blue. Balls represent the amino acids under positive selection. (A) The red balls represent codons selected simultaneously by SLAC, FEL and REL methods, while green balls stand for codons selected by at least two of these methods. (B) Model structure showing the predicted interactions between V3, C2 and C3 in HIV-2 gp125. The non-covalent interaction involves residues Tyr296 and His301 in C2 binding, respectively, to Arg331 and Trp334 in V3, and Phe337 in C3 binding to Phe321 in V3.
(0.88 MB TIF)

Figure S7 Solvent accessible surface area, positive selection and potential N-glycosylation sites in C2-V3-C3 region. (A) HIV-1 alignment (Control dataset). Sites were numbered according to codon env position of HIV-1 HXB2 reference strain. (B) HIV-2 alignment (Control dataset). Sites were numbered according to codon env position of HIV-2 ALI reference strain. Coloured bars represent the amino acids under positive selection and have the same colours (red and green) as the corresponding positions (balls) highlighted in Figure S6. The dark blue stars over the bars correspond to potential N-glycosylation sites conserved along the alignment (present in ≥50% of strains), whereas the light blue stars represent sites only present in less than 50% of sequences.
(1.60 MB TIF)

Table S1 Summary of results for phylogenetic, codon selection and solvent accessibility analysis for C2, V3 and C3 regions of HIV-1 and HIV-2 Control datasets.
(0.04 MB DOC)

Table S2 Positively selected sites detected by SLAC, FEL, REL and/or IFEL in Control HIV-1 and HIV-2 env C2, V3 and C3 regions.
(0.10 MB DOC)

Alignment S1 Alignment of HIV-1 reference sequences used as a Control for the Portuguese HIV-1 dataset. Each sequence is identified by the corresponding GenBank accession number.
(0.04 MB TXT)

Alignment S2 Alignment of HIV-2 sequences used as a Control for the Portuguese HIV-2 dataset. Each sequence is identified by the corresponding GenBank accession number.
(0.02 MB TXT)
