# Supplementary figures and images for: Evolutionary and Structural Features of the C2, V3 and C3 Envelope Regions Underlying the Differences in HIV-1 and HIV-2 Biology and Infection
Source: PLoS One. 2011 Jan 20;6(1):e14548. doi: 10.1371/journal.pone.0014548 (PMC3024314; doi:10.1371/journal.pone.0014548)

A

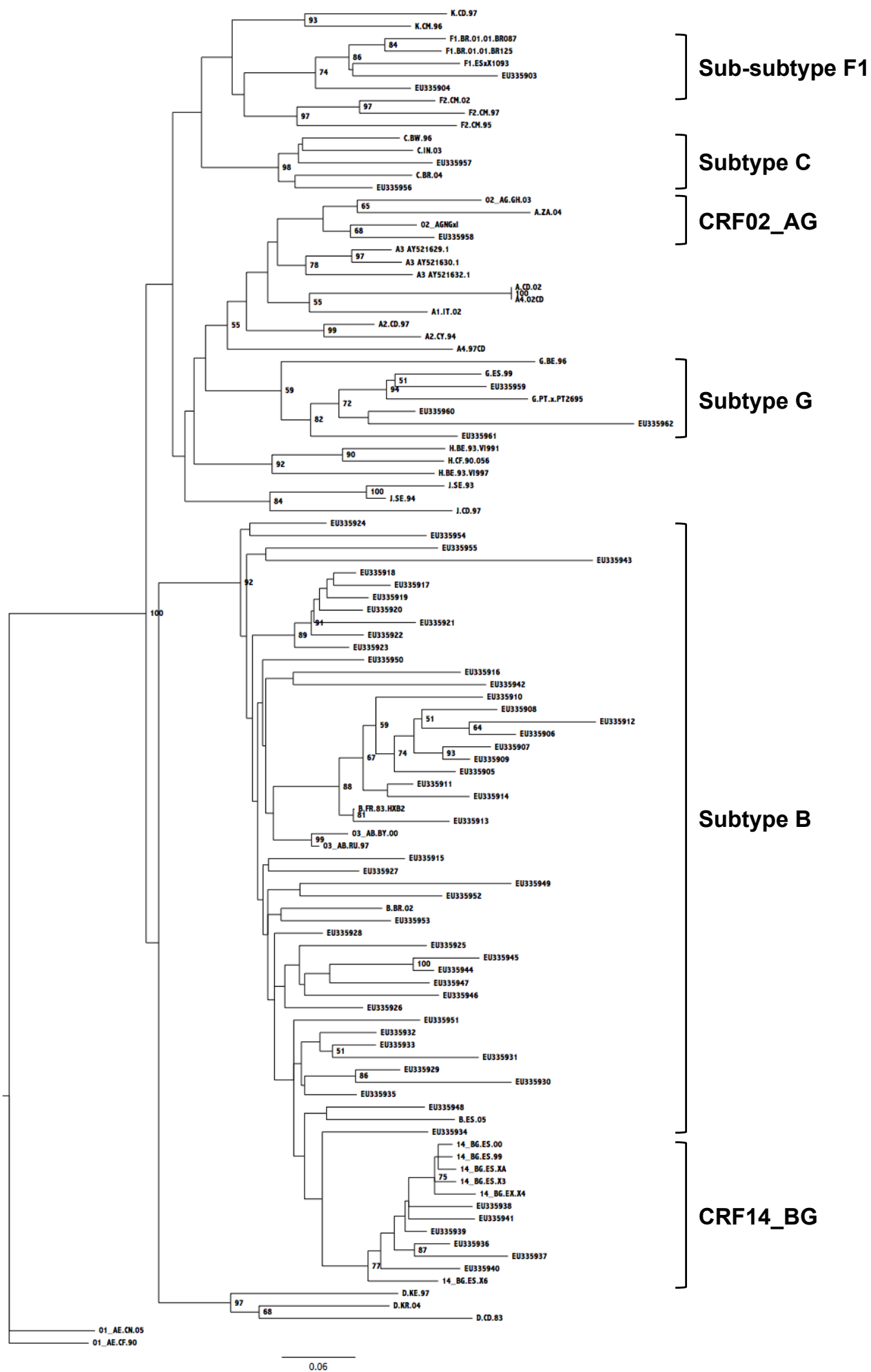

B

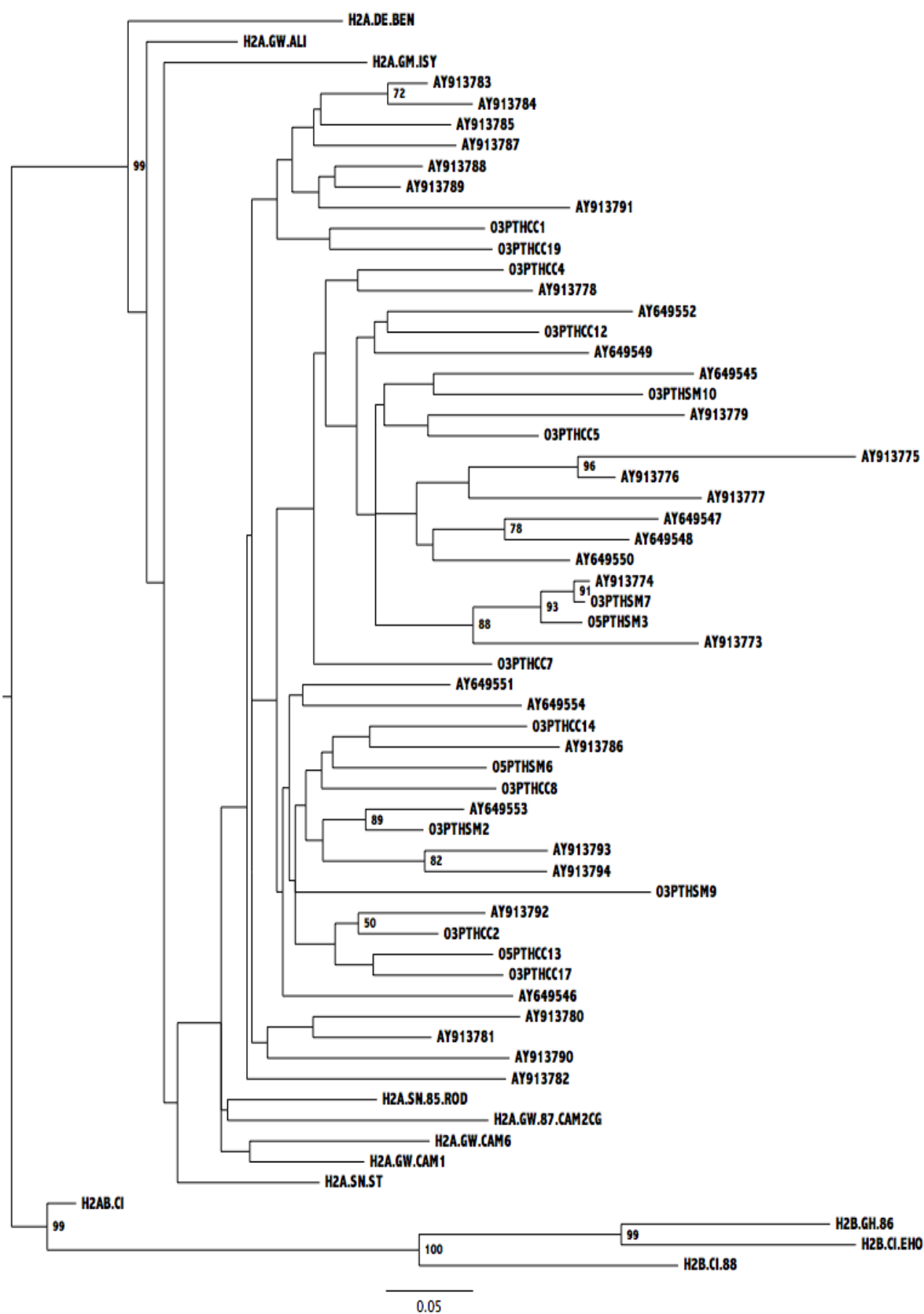

Group A

Supplement: Figure S1 — Genotyping HIV-1(A) and HIV-2 (B) by maximum-likelihood phylogenetic analysis. The phylogenetic trees were constructed using the SPR heuristic search strategy and 1000 bootstrap replications, with reference sequences from HIV-1, under the TVM+G+I evolutionary model (A) and with reference sequences from HIV-2, under the GTR+G+I evolutionary model (B). The bootstrap values (above 50%) supporting the internal branches are shown. The scale bar represents evolutionary distances in substitutions per site. (0.21 MB PDF) [file pone.0014548.s001.pdf]

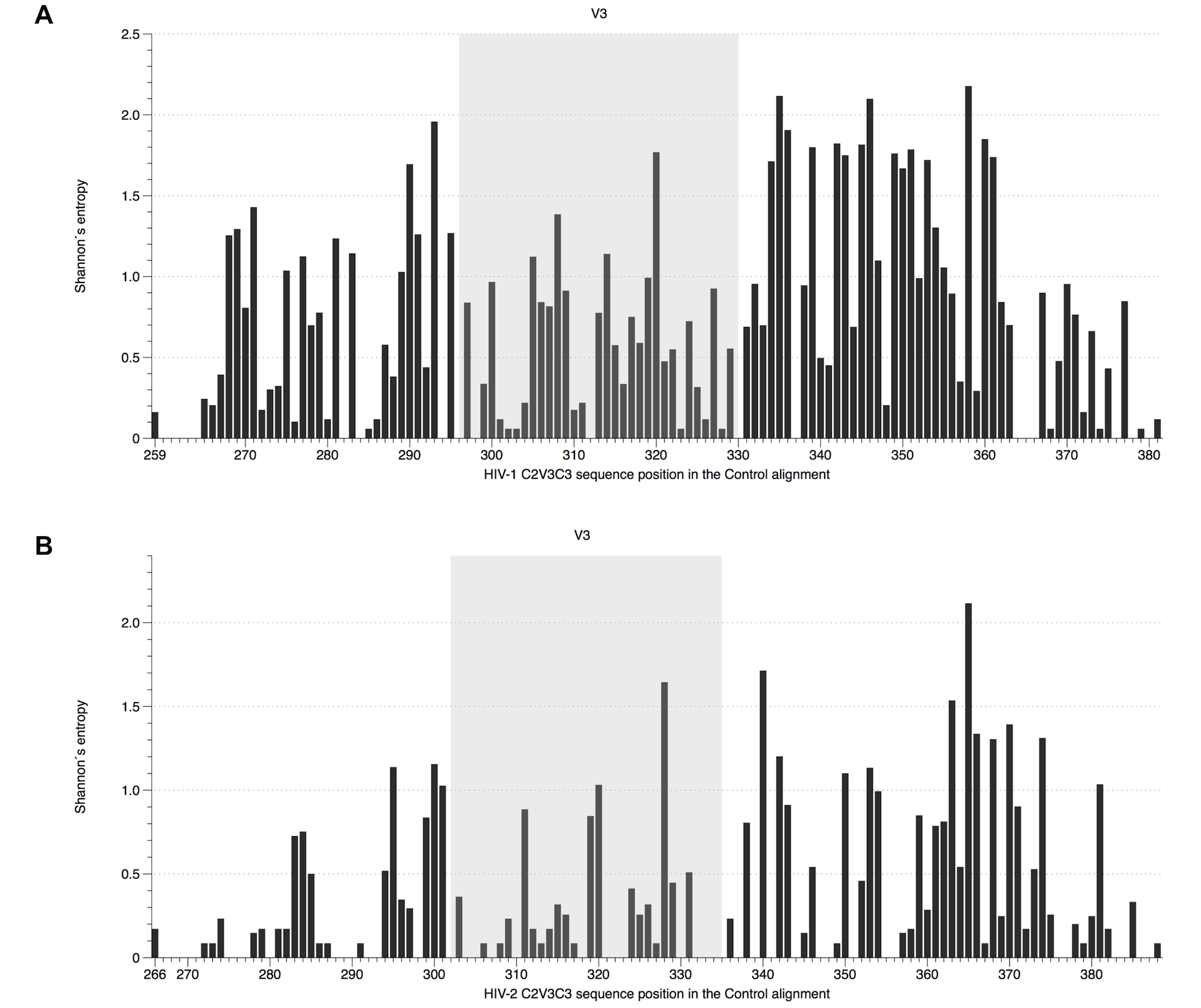

Supplement: Figure S2 — Shannon's entropy of individual amino acids in the C2, V3 and C3 envelope regions in HIV-1 and HIV-2. (A) HIV-1 alignment (Control dataset), sites were numbered according to codon env position of HIV-1 HXB2 reference strain; (B) HIV-2 alignment (Control dataset), sites were numbered according to codon env position of HIV-2 ALI reference strain. (0.96 MB TIF) [file pone.0014548.s002.tif]

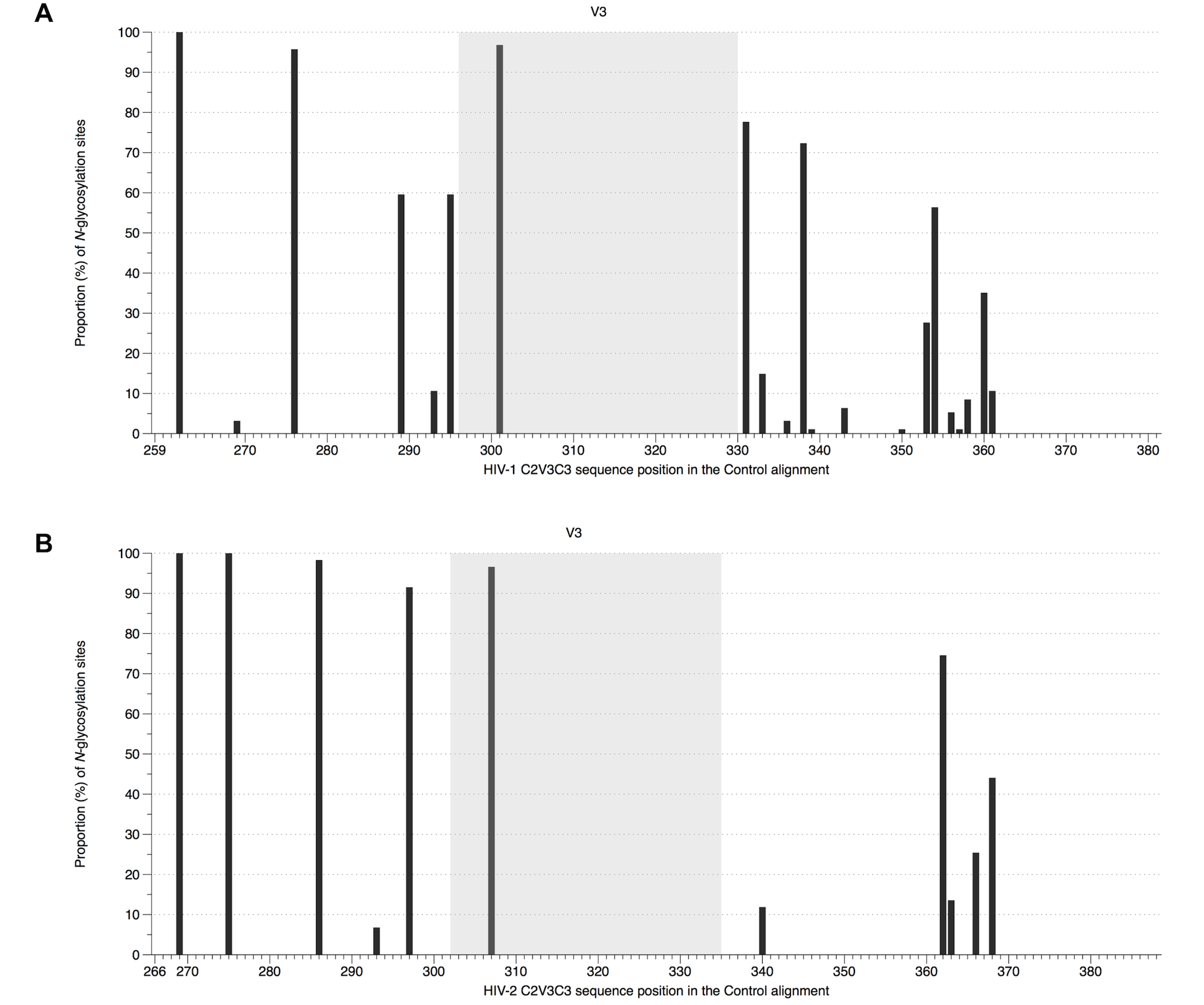

Supplement: Figure S3 — Frequency of N-glycosylation sites in the C2, V3 and C3 envelope regions in HIV-1 and HIV-2. (A) HIV-1 alignment (Control dataset). Sites were numbered according to codon env position of HIV-1 HXB2 reference strain. (B) HIV-2 alignment (Control dataset). Sites were numbered according to codon env position of HIV-2 ALI reference strain. (0.58 MB TIF) [file pone.0014548.s003.tif]

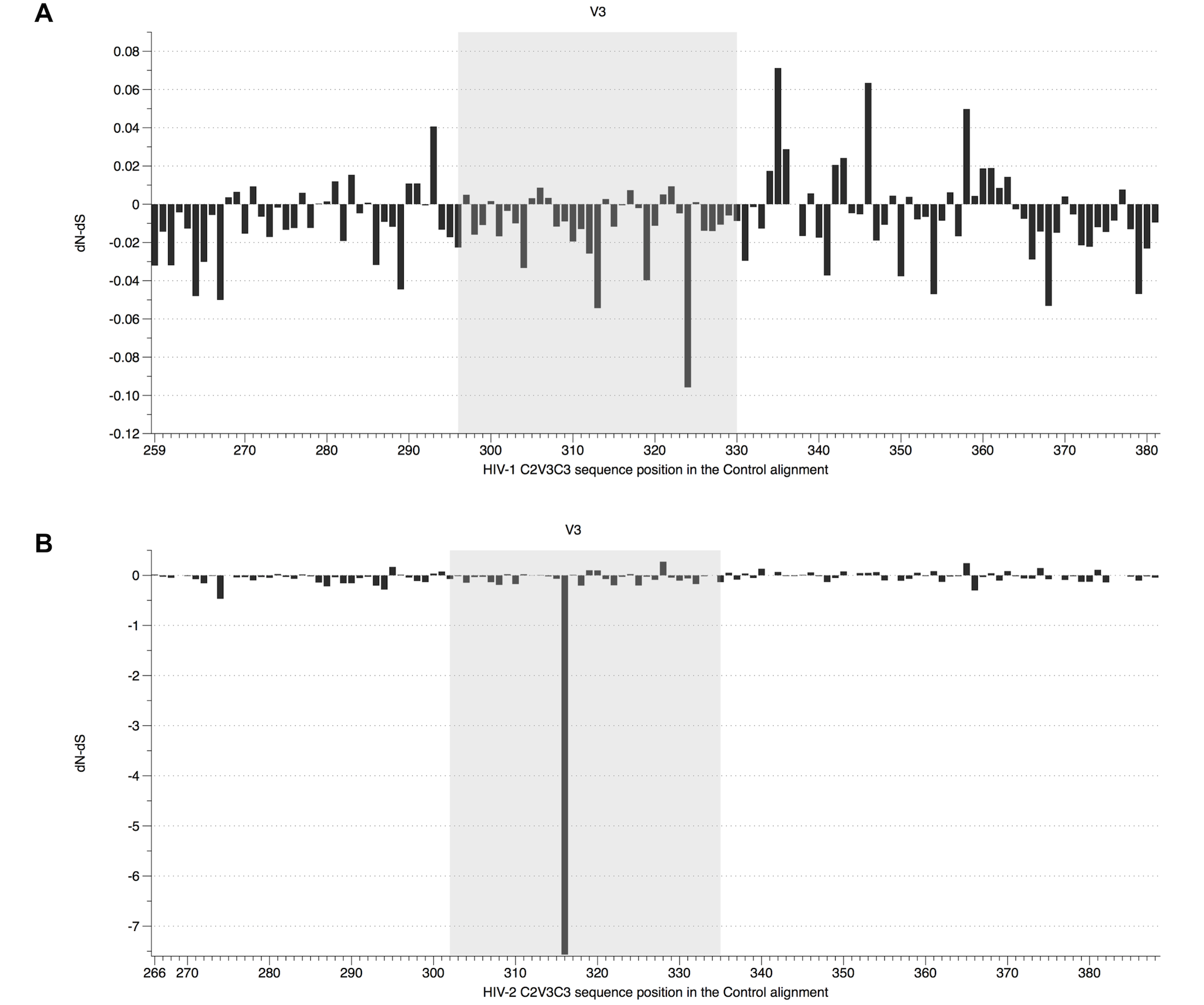

Supplement: Figure S4 — Positive selection in the C2, V3 and C3 envelope regions in HIV-1 and HIV-2. dN-dS values were estimated by FEL and scaled by the total codon tree length. (A) HIV-1 alignment (Control dataset). Sites were numbered according to codon env position of HIV-1 HXB2 reference strain. (B) HIV-2 alignment (Control dataset). Sites were numbered according to codon env position of HIV-2 ALI reference strain. (0.53 MB TIF) [file pone.0014548.s004.tif]

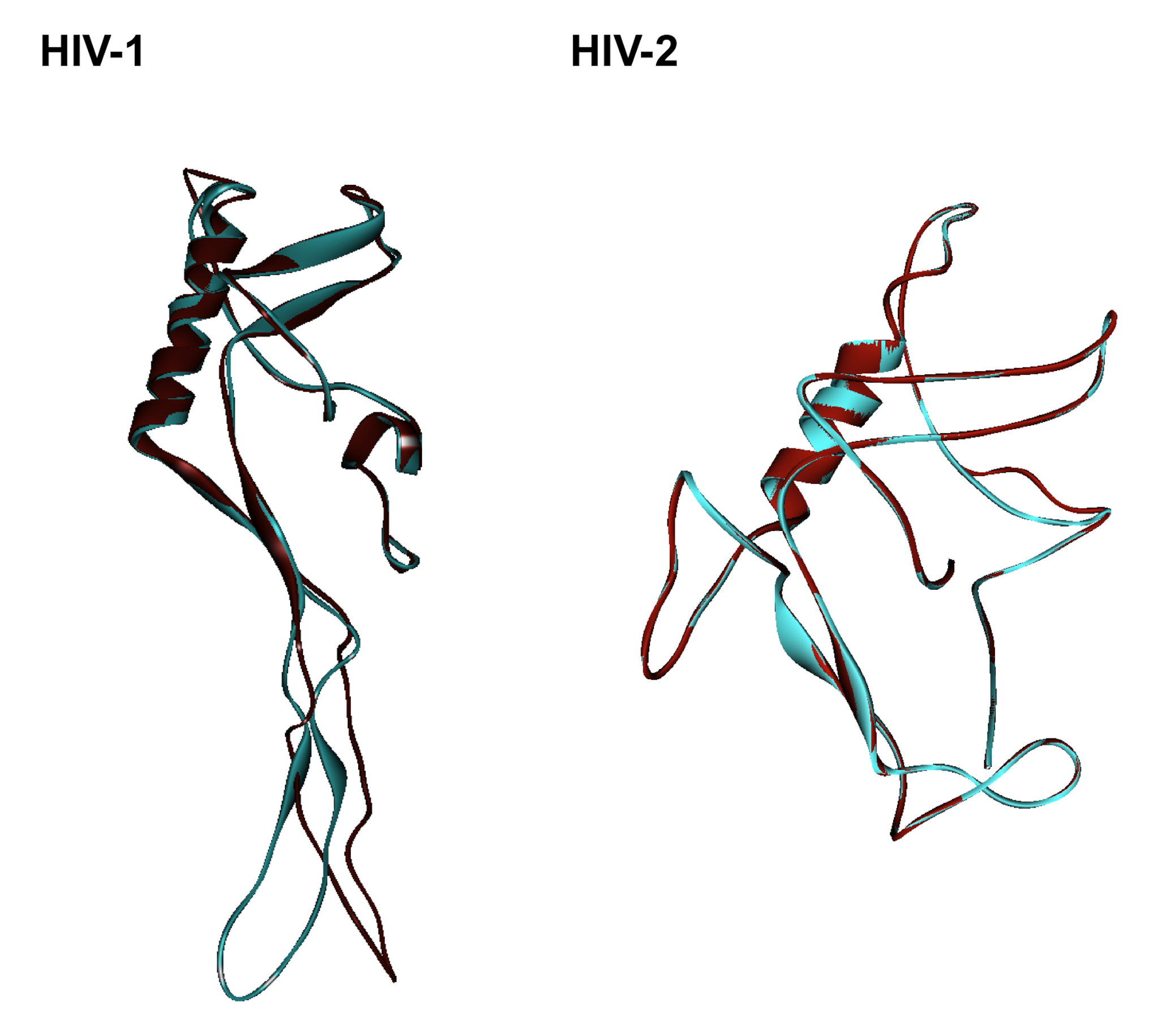

Supplement: Figure S5 — Superimposition of the conformational structures generated by homology modelling of Portuguese and Control C2, V3 and C3 regions of HIV-1 and HIV-2. In the schematics, Portuguese structures are represented in red, and Control structures are in blue. (0.78 MB TIF) [file pone.0014548.s005.tif]

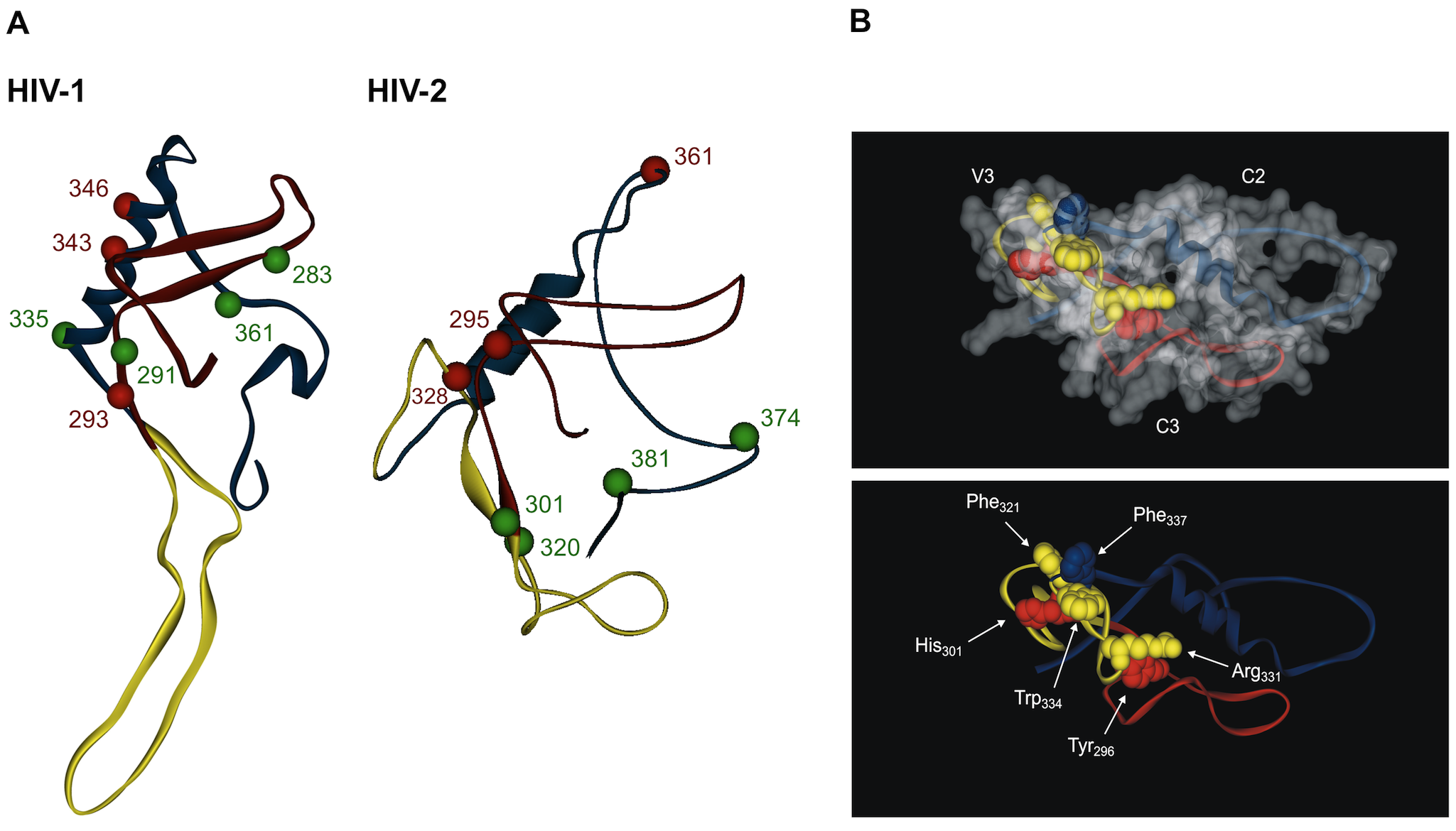

Supplement: Figure S6 — Conformational structure of C2, V3 and C3 envelope regions in HIV-1 and HIV-2. The conformational structure of consensus amino acid sequences derived from the Control datasets was obtained by homology modeling as indicated in “Materials and Methods.” In the schematics, C2 is shown in red, V3 in yellow, and C3 in blue. Balls represent the amino acids under positive selection. (A) The red balls represent codons selected simultaneously by SLAC, FEL and REL methods, while green balls stand for codons selected by at least two of these methods. (B) Model structure showing the predicted interactions between V3, C2 and C3 in HIV-2 gp125. The non-covalent interaction involves residues Tyr296 and His301 in C2 binding, respectively, to Arg331 and Trp334 in V3, and Phe337 in C3 binding to Phe321 in V3. (0.88 MB TIF) [file pone.0014548.s006.tif]

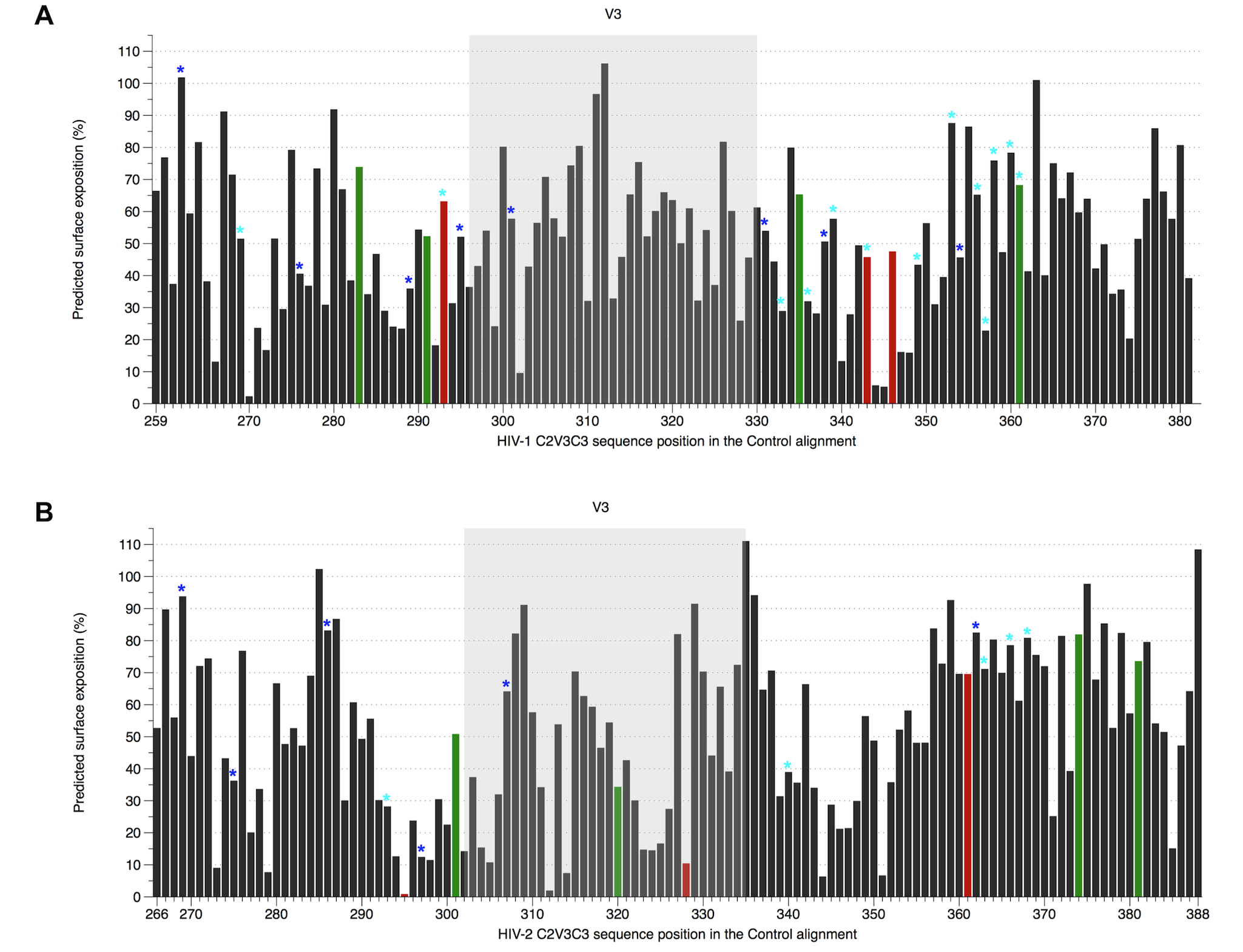

Supplement: Figure S7 — Solvent accessible surface area, positive selection and potential N-glycosylation sites in C2-V3-C3 region. (A) HIV-1 alignment (Control dataset). Sites were numbered according to codon env position of HIV-1 HXB2 reference strain. (B) HIV-2 alignment (Control dataset). Sites were numbered according to codon env position of HIV-2 ALI reference strain. Coloured bars represent the amino acids under positive selection and have the same colours (red and green) as the corresponding positions (balls) highlighted in Figure S6. The dark blue stars over the bars correspond to potential N-glycosylation sites conserved along the alignment (present in ≥50% of strains), whereas the light blue stars represent sites only present in less than 50% of sequences. (1.60 MB TIF) [file pone.0014548.s007.tif]
