# Supplementary material for: Evolutionary and Structural Features of the C2, V3 and C3 Envelope Regions Underlying the Differences in HIV-1 and HIV-2 Biology and Infection
Source: PLoS One. 2011 Jan 20;6(1):e14548. doi: 10.1371/journal.pone.0014548 (PMC3024314; doi:10.1371/journal.pone.0014548)
Supplement: Table S1 — Summary of results for phylogenetic, codon selection and solvent accessibility analysis for C2, V3 and C3 regions of HIV-1 and HIV-2 Control datasets. (0.04 MB DOC) [file pone.0014548.s008.doc]

**Table S1.**

|  | **HIV-1** | **HIV-2** | **P value** |
| --- | --- | --- | --- |
| **Nucleotide diversity**, (mean, [95%CI]) | 0.361 [0.359; 0.364] | 0.221 [0.218; 0.224] | <0.0001 |
| **Entropy**, (mean, [95%CI]) |  |  |  |
| C2-V3-C3 | 0.702 [0.590; 0.813] | 0.353 [0.269; 0.436] | <0.0001 |
| C2 | 0.584 [0.394; 0.775] | 0.244 [0.126; 0.363] | 0.005 |
| V3 | 0.537 [0.382; 0.692] | 0.244 [0.115; 0.373] | 0.002 |
| C3 | 0.900 [0.700; 1.100] | 0.496 [0.344; 0.648] | 0.004 |
| **Entropy values above 1**, (n. sites, (%)) |  |  |  |
| C2-V3-C3 | 34 (27.6%) | 16 (13%) | na |
| C2 | 12 (32.4%) | 3 (8.3%) | na |
| V3 | 4 (11.4%) | 2 (5.9%) | na |
| C3 | 18 (35.3%) | 11 (20.8%) | na |
| **Potential glycosylation sites**, (n. sites, (range) |  |  |  |
| C2-V3-C3 | 7 (5 - 9) | 7 (4 - 10) | na |
| C2 | 3 (1 - 4) | 4 (3 - 5) | na |
| V3 | 1 (0 - 1) | 1 (0 - 1) | na |
| C3 | 3 (2 - 5) | 2 (0 - 5) | na |
| **dN/dS ratio**, (mean, [95%CI]) | 0.649 [0.621, 0.677] | 0.461 [0.427, 0.497] | na |
| **Positively selected sites**, consensus from SLAC/FEL/REL (n. sites) |  |  |  |
| C2-V3-C3 | 3 | 3 | na |
| C2 | 1 | 1 | na |
| V3 | 0 | 1 | na |
| C3 | 2 | 1 | na |
| **Solvent accessibility above 70%** (n. residues) |  |  |  |
| C2-V3-C3 | 27 | 37 | na |
| C2 | 9 | 8 | na |
| V3 | 8 | 8 | na |
| C3 | 10 | 21 | na |

[95%CI] – 95% confidence interval.

P value – P values for the non-parametric Mann-Whitney U test.

na – not applied.
