# Supplementary material for: Evolutionary and Structural Features of the C2, V3 and C3 Envelope Regions Underlying the Differences in HIV-1 and HIV-2 Biology and Infection
Source: PLoS One. 2011 Jan 20;6(1):e14548. doi: 10.1371/journal.pone.0014548 (PMC3024314; doi:10.1371/journal.pone.0014548)
Supplement: Table S2 — Positively selected sites detected by SLAC, FEL, REL and/or IFEL in Control HIV-1 and HIV-2 env C2, V3 and C3 regions. (0.10 MB DOC) [file pone.0014548.s009.doc]

**Table S2**

| **Control HIV-1** | | | | | | | | | | | **Control HIV-2** | | | | | | | | |
| --- | --- | --- | --- | --- | --- | --- | --- | --- | --- | --- | --- | --- | --- | --- | --- | --- | --- | --- | --- |
| **Region** | **Codon** | **SLAC** |  | **FEL** |  | **REL** |  | **IFEL** |  | **Region** | Codon | SLAC |  | FEL |  | REL |  | IFEL |  |
| **C2** | 268 | **1.308** | (0.030) | 0.003 | (0.893) | 0.337 | (0.533) | -0.024 | (0.271) | **C2** | **295** | **1.031** | (0.039) | **0.169** | (0.021) | **1.469** | (1.000) | 0.114 | (0.183) |
|  | 269 | 0.145 | (0.489) | 0.007 | (0.513) | **0.776** | (0.949) | 0.015 | (0.356) |  | 301 | **0.658** | (0.092) | **0.077** | (0.097) | -0.362 | (<0.001) | 0.06 | (0.248) |
|  | 281 | 0.787 | (0.109) | 0.012 | (0.193) | **0.821** | (0.992) | 0.012 | (0.399) |  |  |  |  |  |  |  |  |  |  |
|  | 283 | 0.775 | (0.118) | **0.020** | (0.023) | **0.837** | (0.999) | 0.018 | (0.158) |  |  |  |  |  |  |  |  |  |  |
|  | 290 | **1.645** | (0.024) | 0.013 | (0.574) | -0.033 | (0.185) | -0.015 | (0.535) |  |  |  |  |  |  |  |  |  |  |
|  | 291 | **1.103** | (0.012) | 0.013 | (0.201) | **0.824** | (0.959) | 0.022 | (0.137) |  |  |  |  |  |  |  |  |  |  |
|  | **293** | **2.672** | (<0.001) | **0.060** | (0.003) | **0.817** | (0.948) | **0.054** | (0.021) |  |  |  |  |  |  |  |  |  |  |
| **V3** | 305 | **1.034** | (0.062) | 0.007 | (0.712) | 0.593 | (0.768) | -0.008 | (0.661) | **V3** | 320 | 0.498 | (0.229) | **0.099** | (0.099) | **1.197** | (0.990) | **0.28** | (0.007) |
|  | 306 | **0.972** | (0.035) | 0.011 | (0.366) | -0.046 | (0.262) | -0.010 | (0.399) |  | **328** | **1.357** | (0.024) | **0.273** | (0.007) | **1.011** | (0.999) | 0.145 | (0.191) |
|  | 314 | **0.943** | (0.055) | 0.005 | (0.651) | 0.758 | (0.931) | -0.009 | (0.496) |  |  |  |  |  |  |  |  |  |  |
|  | 317 | **0.706** | (0.027) | 0.008 | (0.209) | -0.026 | (0.486) | **0.018** | (0.075) |  |  |  |  |  |  |  |  |  |  |
|  | 322 | 0.576 | (0.146) | **0.014** | (0.041) | -0.041 | (0.463) | 0.005 | (0.263) |  |  |  |  |  |  |  |  |  |  |
| **C3** | 332 | 0.184 | (0.420) | 0.003 | (0.723) | -0.239 | (0.057) | **0.031** | (0.047) | **C3** | 340 | 0.77 | (0.177) | 0.132 | (0.299) | **0.756** | (0.968) | 0.191 | (0.451) |
|  | 334 | **2.069** | (0.001) | 0.030 | (0.106) | 0.591 | (0.773) | 0.038 | (0.123) |  | 342 | 0.302 | (0.378) | 0.068 | (0.334) | **0.851** | (0.997) | 0.095 | (0.397) |
|  | 335 | **3.582** | (<0.001) | **0.102** | (<0.001) | 0.584 | (0.761) | **0.075** | (0.006) |  | 346 | 0.544 | (0.129) | **0.059** | (0.067) | -0.245 | (<0.001) | 0.02 | (0.553) |
|  | 336 | **1.927** | (0.002) | 0.029 | (0.185) | 0.580 | (0.761) | 0.023 | (0.350) |  | 353 | 0.605 | (0.187) | 0.049 | (0.470) | **0.842** | (0.999) | 0.018 | (0.840) |
|  | 342 | **0.465** | (0.026) | 0.011 | (0.614) | 0.307 | (0.506) | -0.020 | (0.250) |  | **361** | **0.602** | (0.091) | **0.084** | (0.021 | **0.404** | (1.000) | 0.042 | (0.358) |
|  | **343** | **0.360** | (0.045) | **0.030** | (0.004) | **0.837** | (1.000) | **0.041** | (0.012) |  | 363 | -0.22 | (0.706) | -0.022 | (0.821) | **0.759** | (0.969) | 0.004 | (0.976) |
|  | **346** | **0.767** | (<0.001) | **0.052** | (0.001) | **0.961** | (0.994) | **0.053** | (0.004) |  | 365 | **1.499** | (0.043) | 0.244 | (0.200) | -0.338 | (0.557) | 0.218 | (0.355) |
|  | 349 | **0.273** | (0.095) | 0.004 | (0.781) | 0.400 | (0.594) | 0.005 | (0.791) |  | 368 | -0.17 | (0.702) | 0.043 | (0.581) | **0.837** | (0.998) | 0.005 | (0.962) |
|  | 351 | 0.037 | (0.510) | 0.005 | (0.657) | **0.770** | (0.943) | -0.005 | (0.696) |  | 370 | 0.639 | (0.172) | 0.085 | (0.287) | **0.85** | (0.999) | 0.045 | (0.667) |
|  | 356 | **0.183** | (0.097) | 0.005 | (0.531) | -0.196 | (0.143) | -0.002 | (0.887) |  | 374 | 0.831 | (0.102) | **0.145** | (0.095) | **0.926** | (0.999) | **0.245** | (0.065) |
|  | 358 | 0.164 | (0.254) | **0.044** | (0.003) | 0.708 | (0.885) | **0.083** | (0.002) |  | 381 | **0.882** | (0.023) | **0.111** | (0.009) | -0.339 | (<0.001) | **0.126** | (0.013) |
|  | 360 | 0.263 | (0.122) | 0.010 | (0.507) | 0.544 | (0.730) | **0.043** | (0.087) |  |  |  |  |  |  |  |  |  |  |
|  | 361 | **0.477** | (0.005) | 0.015 | (0.247) | **0.796** | (0.960) | **0.041** | (0.032) |  |  |  |  |  |  |  |  |  |  |
|  | 363 | **0.262** | (0.012) | 0.010 | (0.101) | 0.061 | (0.661) | 0.000 | (0.980) |  |  |  |  |  |  |  |  |  |  |
|  | 370 | **0.169** | (0.077) | 0.004 | (0.587) | -0.103 | (0.329) | 0.007 | (0.432) |  |  |  |  |  |  |  |  |  |  |
|  | 377 | **0.243** | (0.025) | 0.007 | (0.289) | -0.072 | (0.392) | 0.000 | (0.961) |  |  |  |  |  |  |  |  |  |  |

Codon – codons selected under 10% level of significance (SLAC, FEL and IFEL), or above a Bayes Factor of 50 (REL) and numbered according to codon *env* position of HIV-1 HXB2 for HIV-1 dataset or of HIV-2 ALI for HIV-2 dataset. Codons selected simultaneously by SLAC, FEL and REL methods are bold and underlined.

SLAC, FEL and IFEL – the first numbers are the dN-dS difference for each site scaled by the total codon tree length, the numbers in parenthesis show P-values for corresponding test of non-synonymous rate being superior to synonymous rate;

REL - the first numbers are the expected posterior dN-dS difference for each site scaled to the total codon tree length, the number in parenthesis show the posterior probability of non-synonymous rate being superior to synonymous rate;

Bold dN-dS differences correspond to significant P-values or posterior probabilities;
